# Teaching to prepare undergraduate nursing students for palliative care: nurse educators’ perspectives

**DOI:** 10.1186/s12912-023-01493-5

**Published:** 2023-09-27

**Authors:** Ramona Schenell, Jane Österlind, Maria Browall, Christina Melin-Johansson, Carina Lundh Hagelin, Elin Hjorth

**Affiliations:** 1 The Sahlgrenska Academy, Institute of health and care sciences, University of Gothenburg, Box 457, Gothenburg, 405 30 Sweden; 2 Administration for the elderly, nursing and care, Department of Quality and development, The City of Gothenburg, Box 334, Västra Frölunda, 42123 Sweden; 3 Department of Health Care Sciences, Palliative Research Centre, Marie Cederschiöld University, Box 11189, Stockholm, 100 61 Sweden; 4 Faculty of caring sciences, work life and social welfare, department of caring sciences, University of Borås, Borås, Sweden; 5 Affiliated to Dep of Oncology, Inst of Clinical Sciences, Sahlgrenska Academy, University of Gothenburg, Gothenburg, Sweden; 6 Department of Health Care Sciences/Nursing Sciences, Mid Sweden University, Kunskapens väg 8, 831 25, Östersund, Sweden; 7 Department of Neurobiology, Care Sciences and Society, Division of Nursing, Karolinska Institutet, Stockholm, Sweden

**Keywords:** Nurse education, Teaching approach, Student-centred education, Pedagogy, Palliative care, End of life care

## Abstract

**Background:**

Education in palliative care for undergraduate nursing students is important for the competence of general nurses. Newly graduated nurses have reported challenges in coping with their own emotions when encountering dying persons. They express a wish for more education before they graduate, particularly in psychosocial and existential areas, such as having difficult conversations and supporting grieving persons. Despite awareness of the importance of palliative care education for nurses, there is a lack of knowledge on how to effectively convey this knowledge to students. The aim of the present study was to explore how teaching to prepare undergraduate nursing students for palliative care practice is conducted in Sweden.

**Methods:**

Educators from 22 Bachelor of Science nursing programmes in Sweden were interviewed about how they conducted palliative care education, with a focus on teaching situations that have been successful or less successful. The interviews were transcribed and analysed using qualitative inductive content analysis.

**Results:**

Educators described that they play a crucial role in preparing undergraduate nursing students to face death and dying and to care for persons at the end of life. In the main theme, “Transforming person-centred palliative care into student-centred education”, educators described how they incorporated the person-centred palliative approach into their teaching. Educators used a dynamic style of teaching where they let the students’ stories form the basis in a co-constructed learning process. The educators trusted the students to be active partners in their own learning but at the same time they were prepared to use their expert knowledge and guide the students when necessary. Discussion and reflection in small groups was described as being essential for the students to achieve a deeper understanding of palliative care and to process personal emotions related to encountering dying and grieving individuals.

**Conclusions:**

This study suggests that palliative care education for undergraduate nursing students benefits from teaching in smaller groups with room for discussion and reflection. Furthermore, gains are described relating to educators taking the role of facilitators rather than traditional lecturers, being flexible and ready to address students’ emotions. Educators also draw on their experiences as palliative care nurses in their teaching practices.

## Background

Nurse educators play an important role in supporting and encouraging undergraduate nursing students to prepare for caring for patients at the end of life and to encounter death and dying [1]. Society is facing an ageing population and a growing incidence of older people with cognitive impairment and multimorbidity, which implies the need to integrate palliative care into health systems [2]. Palliative care is an approach that improves the quality of life of patients and families who face problems associated with chronic as well as life-threatening illness. This means working with a focus on early identification, the prevention and relief of suffering, along with the treatment of pain and other physical, psychosocial and/or spiritual problems [3]. Palliative care is active and interdisciplinary, focusing on the patient, the family and the surrounding community, and should be provided in most caring settings, across all stages of life-threatening illness, and for patients of all ages [4]. To promote quality of life and a good death for each person, the palliative care approach is person-centred and takes its starting point from the person’s own narratives about their needs and preferences. The healthcare professional should strive to understand how the person is experiencing the situation and what factors contribute to meaning, dignity, and relief from distress by engaging in a genuine dialogue with the individual. Based on an assemblage of the ill person’s experiential knowledge and the healthcare professional’s professional and scientific knowledge, the care should be co-created to meet the persons preferences [5]. To enable a person-centred approach when meeting the ill person, family, and colleagues in palliative care, registered nurses need competencies in collaboration, communication, and leadership. Adopting a holistic view, including clinical, psychosocial, spiritual, and ethico-legal competency, is also required [6].

Information and good communication along the illness trajectory is crucial in palliative care, and palliative care nurses have reported that having the courage to be present and confirming, taking time and not trying to ‘solve’ existential issues, are important factors in conversations with persons close to death. However, conversations about existential issues are sometimes described by nurses as a heavy burden that drain them of energy and trigger anxiety. In particular, newly trained nurses report feeling naked and lacking appropriate tools when faced with existential issues and desire more reflection, training, and education [7]. Similar challenges among newly graduated nurses, when caring for dying persons, have been found in other studies, and these suggest that more training is needed before graduation, for example, more skills in dealing with difficult conversations are necessary [8, 9]. The lack of tools and the triggering of one’s own emotions might explain why only a minority of nurses report that they talk to seriously ill persons and their families about death, despite acknowledging the importance of supporting them with their emotional coping [10].

Internationally, the principles of palliative care are not included in all undergraduate nursing curricula [4, 11]. In Sweden, all universities incorporate education about palliative care in some way, but it is not always a prioritised topic [12]. Students’ attitudes and preparedness toward caring for dying persons can be improved within the education programme [13]. Changes in attitudes, to some extent, depend on the length of the theoretical component of the education programme, in that undergraduate nursing students who receive longer courses of palliative care education change positively in their attitudes toward providing care to dying persons [14, 15]. To achieve positive progress, undergraduate nursing students need continuous support and opportunity to reflect and discuss both end-of-life care and death throughout their education [16].

Despite the existing knowledge about the importance of education in palliative care for nurses, there is a lack of knowledge in how to provide this to students [17]. A systematic review identified 16 articles examining the impact of various teaching methods employed in palliative care education. These showed that different pedagogical methods, such as simulations, lectures, films, problem-based learning and game interventions, all had a positive effect on learning [18]. In order to obtain a clearer picture of how educators prepare undergraduate nursing students for palliative care, this present study took its origin from the educators’ perspective. The aim of the study was to explore how teaching in palliative care was conducted in Sweden.

## Methods

This study is part of a larger study with the overall aim to investigate the extent, content and pedagogical methods of palliative care education for undergraduate nursing students [12]. This specific study employed a qualitative design with individual telephone interviews with nurse educators, inspired by the critical incident technique. The critical incident technique is used for ascertaining the general aims of a specific activity being studied and to collect information about the behaviours of persons involved in the activity [19].

### Study settings

In Sweden, registered nurses complete a three-year bachelor’s programme in nursing before being registered as nurses. Today, 25 universities in Sweden are responsible for providing nursing education at the basic level. Although the Swedish National Agency for Higher Education has defined national learning goals for nursing education, education specific to providing palliative care varies between the universities, in its content and ECTS credits [12].

### Participants

At the time of the data collection there were 24 universities providing Bachelor of Science nursing programmes in Sweden. All 24 universities were contacted. Educators with a responsibility for providing education in palliative care for undergraduate nursing students were invited to participate. The educators were identified through the universities’ web pages or by contacting the person responsible for the relevant department. An e-mail was sent with information about the study along with an invitation to participate. A week later, a follow-up email was sent requesting participation. Twenty-two universities agreed to participate and were represented by one educator each. Twenty participants were women and 2 were men and their length of experience as educators varied between 1 and 36 years (md = 12).

### Data collection

Interviews were performed by telephone, audio-recorded and lasted between 14 and 60 min (md = 30). The interview guide was comprehensive and covered several areas in palliative care education. Previous results from the interviews have been published elsewhere [12]. However, this specific study focused on two questions in the interview guide that have not been analysed before. To gain rich descriptive examples from the educators’ work experiences, two main questions, inspired by the critical incident technique, were asked: *Can you describe a situation in palliative care education that was particularly positive for both you and the students?* and *Can you describe a situation in palliative care education that was less positive for both you and the students?* To deepen the understanding about the general aims and the behaviours of those involved in the teaching situation four follow-up questions were asked to each main question; *what happened, what did you think, how did you feel, and what influenced the outcome for you and the students?* To further deepen the educators’ narratives and the understanding about the situations, questions like *can you elaborate on that a bit more?* and *what do you mean?* were asked. The interviews were performed by four of the researchers, all with clinical experience of palliative care as well as experience of research and teaching in this area.

### Data analysis

The interviews were transcribed verbatim and inductive latent content analysis was used [20]. The first and last author read the text several times to gain a sense of the whole and identified meaning units with aspects relating to each other through content and context. The meaning units were labelled with codes which were compared for differences and similarities and sorted into tentative manifest categories. Through a process of interpretation, asking ‘What is this about?’, and returning to the transcribed texts, the content of the categories was abstracted into themes and subthemes which comprised the latent content of the data. Further interpretation of the meaning of the themes and subthemes resulted in a suggestion of a main theme, illuminating the comprehensive interpretation of the result. At this stage, all co-authors critically reviewed the results, which were read, reflected on, and discussed recurrently until consensus about the interpretations was reached.

## Results

The findings are presented as an all-permeating main theme, *Transforming person-centred palliative care into student-centred teaching*, and the two themes, *Focusing on emotional preparation* and *Promoting in-depth learning by reflection*, with their respective subthemes (Table 1).

**Table 1:** Presentation of main theme, themes, and subthemes

MAIN THEME	TRANSFORMING PERSON-CENTRED CARE INTO STUDENT-CENTRED TEACHING	
**THEMES**	Focusing on emotional preparations	Promoting in-depth learning by reflection
**SUBTHEMES**	Conduct confrontational teaching	Perform dynamic teaching
	Prepare for students’ reactions	Give responsibility to the students
	Understand the importance of group dynamics	Connect theory to practice

### Transforming person-centred palliative care into student-centred teaching

The main theme, *Transforming person-centred palliative care into student-centred teaching*, was created when several of the core features of the person-centred palliative care, such as listening to the narrative, co-creation of the care, and seeing the person as capable, became visible in the analysis. The educators in the study conveyed a way to teach students in a similar manner, as they, as nurses themselves, cared for ill persons. Throughout the interviews, educators described how they adapted their teaching to the needs of the students and that the starting point in teaching often was the students’ own stories. In successful examples of teaching situations, the student-centred approach allowed for the students to reflect upon their own feelings about dying and grief in a safe education environment. This also allowed the students to reach a deeper understanding as they were seen as being capable persons who could seek their own knowledge, at the same time as they were being guided by the expertise of experienced nurse educators, in a process of co-created learning.

### Focusing on emotional preparations

According to the educators, palliative care education needs to focus on getting the students emotionally equipped for working in palliative care. The educators emphasised that caring for dying persons and their relatives can be challenging, especially for younger students with limited experiences of dying and grief. It was therefore seen as important to prepare the students emotionally to face, not only patients’ and relatives’ feelings, but also their own. The importance of giving the students tools to cope with their emotions was shown throughout the data, as all interviewees described teaching situations, both successful and less successful, with a focus on the emotional and existential aspects of palliative care.

### Conducting confrontational teaching

To emotionally prepare students for palliative care and increase awareness of their own feelings, thoughts, fears, and attitudes, the educators described it as being necessary to confront the students with their feelings about dying and grief, even though this could be emotionally challenging for them. To prepare the students, educators used strategies such as discussing patient cases, talking about existential issues, imagining what it would be like to live with a life-limiting illness, and visiting the crematorium. Being confronted with the emotional aspects of palliative care affected the students and some became upset and sad. According to the educators, these occasions lead to important insights that better equip the students for their future professional role as registered nurses. The educators also described that such confrontational teaching mostly resulted in pleasant moments where the students reflected together on life and death and how difficult, beautiful, and strange it could be. Several educators described that they have had students who came back to them later, saying that these conversations were the most difficult but also the most important during the entire nursing education programme.

Although the educators believed that it is necessary for the students to be confronted by and reflect upon their feelings about death, not all students took part in these discussions, as many elements of this part of the programme were voluntary. There was also an awareness among the educators that some students were experiencing their own grieving process and they therefore did not force them to participate in these discussions.

*We can never know what we have awakened (through the education). But at the same time, soon they will start working and it is important to provide them with tools so they dare to face these situations and stay present in them. And to have the tools to engage in conversations and manage the various emotional responses that arise.* (Educator 1)

### Prepare for students’ reactions

The educators were aware that palliative care education could be difficult for the students and prepared themselves to cope with various situations that may arise during lectures and seminars. If a student became upset or sad, educators described that they, for example, always had tissues handy, and tried to be vigilant to what was happening, paused the lesson and waited for the distressed student to become calm. Another strategy to cope with students’ reactions was to guide the student group to support and comfort each other and by doing so create a common understanding that it was permissible to show emotions. If a student became upset and left the room, the educators considered themselves responsible for speaking to the student afterwards or facilitating support via the student health service. One strategy to capture reactions in larger student groups was having two educators in the classroom, one who focuses on giving the lecture and one who focuses on the students and who helped to adapt the lecture according to their reactions if necessary. Teaching situations described as being less successful and more stressful for the educators were when they were unable to support students who reacted emotionally during lessons. It was described as being challenging to balance support for individuals while at the same time focusing on the learning of the whole student group. If an upset student left a lecture accompanied by many others, it was difficult for the educators to provide support afterwards, as they might not be able to identify who the student was. One educator even feared that a lack in providing emotional support when needed could cause students to drop out of their education programme. Although being prepared for students’ reactions was seen as a natural element in palliative care teaching, the balance between teaching and providing emotional support could cause extra pressure on the educators.

*Last time we had that existential seminar, there was a guy who wanted to talk about euthanasia the whole time. Everyone had their questions, and he was just hung up on euthanasia. And I think that he had thoughts about ending his life. And to try to balance his anxiety, which I think it was, and still make room for the other students. It didn’t turn out very well.* (Educator 2)

### Understand the importance of group dynamics

For the students to approach their feelings about palliative care and talk about existential questions, they need to feel safe with each other. The educators emphasised how facilitating collaboration and dialogue within the student group could have a major impact on how the lesson turned out. Group dynamics are difficult to influence, according to the educators, sometimes the whole student group was active in discussions, while other groups had no commitment to participate. To facilitate deeper discussions, educators highlighted the importance of holding discussions in smaller groups, of around 7–10 students.

The educators testified that they had been involved in situations where one or two students influenced the whole group and created an unpleasant atmosphere, for example, based on different opinions between students or between educators and students. The educators emphasised that it was important to try to sort out such conflicts when they arose. In the examples highlighted as negative, the educators failed to turn the discussion into something constructive, and the atmosphere remained unpleasant. This left the educators with a sense of frustration and failure, and several of them described how they carried these situations with them and questioned their competence as teachers.

*It’s largely dependent on the group as a whole, how they affect each other, because sometimes you have groups where you feel ‘God this turned out well!’ They are sharing, are generous with themselves and are receptive, but with other groups, you can’t quite reach them in the same way.* (Educator 3)

### Promoting in-depth learning by reflection

The educators strived for in-depth learning where the students reach a deeper understanding about palliative care through reflection. Learning on a deeper level was described as being able to understand the relation between theory and practice and to gain insights into how to use this knowledge in various patient care situations. To promote reflection, the educators described how they took on the role of facilitator and, instead of giving “the right answers”, took part in and encouraged the sharing of knowledge and experiences. The boundaries between teacher and student, expert and novice, were, in these cases, blurred and co-created learning, through reflection, emerged.

### Perform dynamic teaching

The educators described positive teaching situations where they left the traditional lecture structure, with students as passive recipients, in favor of a more dynamic teaching method, where students became actively engaged. This required the educator to be responsive and compliant, but also capable of directing and balancing the discussions. A teaching session that began as a whole-class lecture could, for example, be redirected to discussions in small groups to take advantage of emerging experiences or to calm upset feelings. With this approach, no teaching session was the same and it could be difficult for the educators to predict exactly what would happen during the lesson. This was not described as a problem, rather, as a conscious choice to create space for the students to reflect and provide conditions for in-depth learning. Another example of dynamic teaching was to be available in the classroom during breaks to catch questions and reflections. The educators also expressed willingness to develop and refine the teaching by experimenting and making changes based on their own experiences and the students’ feedback and evaluations.

*A lot is required of one as a nurse educator and it is also required that you somehow dare. You don’t know, it’s the same way as with patients, you don’t know what you’re facing. It is the same with seminars and discussions with students. You also let them be – be the ones driving the discussion, and just come in in some way and lead and try to carry it forward. Then I think it is best, when you feel that you actually dare to let go and just be there and let them take care of this. It can turn out really well.* (Educator 3)

### Give responsibility to the students

The educators described that learning could deepen if the students were given responsibility to seek information by themselves before coming to class, in the so-called flipped classroom. Preparations such as watching recorded lectures, answering study assignments, reading study literature, or working with patient cases could be done individually or in groups. When later attending the teaching session, the students were described to arrive at a deeper level of knowledge as they were getting new perspectives from each other. This required that the educators let the students take an active part, but at the same time were prepared to step in and lead discussions if needed.

According to the educators, most students in palliative care education were prepared to take on the responsibility for their own learning. The educators described students as being curious, willing to share their experiences, empathetic, and receptive to others’ stories. Many students also spent time on study assignments and attended seminars, although they were not always mandatory. However, there were students who did not prepare themselves and questioned why they should come to a seminar if it was not mandatory or if the knowledge gained there was not to be tested in an exam. According to the educators, these students risked missing out on opportunities for in-depth learning and thus on important knowledge that they need in their future professional roles as registered nurses.

*Seminars are always good, our students at least feel that it is there, in seminars, they learn. Given that everyone is prepared.* (Educator 4).

### Connecting theory to practice

The educators described that certain theoretical knowledge could be difficult to relate to and therefore difficult for the students to comprehend. To facilitate the understanding of theory and reach a deeper level of knowledge, the students need to connect theory and practice. This could be achieved, for example, when students reflected on experiences from clinical training in relation to the course objectives or practiced theoretical knowledge on a simulation manikin or in role play. The educators’ own experiences were also described as being important, as the educator could then bridge theories in palliative care with practical examples from their own practice. This required that the educators had good clinical anchoring and spoke in a way that clearly positioned such theories in relation to practice. Educators could also diminish the students’ concerns about their future professional role by sharing their own experiences of working in palliative care.

*I firmly believe in linking the theories with practical examples […] I think there is a much greater chance that they will retain the knowledge if they get these concrete examples that clarify the theories and make them understand the connection.* (Educator 5)

## Discussion

The main theme, *Transforming person-centred palliative care into student-centred teaching*, describes how educators have brought the person-centred palliative care approach into palliative care education. In person-centred care, the narrative is the first step in establishing a partnership between the ill person and the caregiver [21]. In this study, the educators described how the students’ narratives often formed the basis for a co-created learning process where the educators trusted the students to be active partners in their own learning. The educators did not abdicate from their role as experts, but, instead of providing all the right answers, they made the students search for information and share experiences with each other, while at the same time they were prepared to intervene and guide the discussions when needed.

According to the hermeneutic philosopher, Ricoeur, a human is first to be understood as a capable being [22]. Looking at palliative care through a hermeneutic lens, there is no contradiction between understanding the ill person as both suffering and capable [23]. The educators in the present study did not only trust the students to be active partners in their own learning, but also trusted their capability to cope with their own emotional reactions and suffering when being confronted with questions about death and dying. Students becoming emotional, and perhaps crying during class, was not seen as a goal in itself. However, students approaching their own feelings was described as being necessary to prepare them for their future professional roles as nurses in palliative care. Previous research has shown that first-year nursing students have concerns and fears about not being able to meet and cope with their own emotions when caring for dying persons [16]. In line with the educators’ experiences in the present study, Poultney et al. highlight the need to confront undergraduate nursing students’ own feeling about death and that this should be achieved within the protection of the classroom [1]. When asked to describe teaching situations, all of the interviewed educators mentioned existential and emotional aspects, which illustrates that psychosocial care is an important part of palliative care. Nevertheless, a systematic review reported that the psychosocial domain, with emotional support, was the most prominent unmet care need of people living with advanced cancer and their informal caregivers. Unmet care needs were associated with their symptoms, anxiety, and quality of life [24]. This highlights the importance of preparing undergraduate nursing students in psychosocial care for the sake of both patients and themselves. Confronting students with death and dying places high demands on the educator’s ability to cope with emotional reactions. In the present study the educators were prepared to manage these situations and spoke calmly about them. The fact that the educators had strategies for encountering upset emotions without becoming distressed themselves might originate from their own experiences as nurses in palliative care. As such, they are familiar with providing emotional support and comfort, and this may explain why it seemed natural for them to do the same in their role as educators. To conclude, although the educators trusted the students’ capability when being confronted with emotional aspects of care, they felt responsible for providing support when needed.

The educators in the present study highlighted large group lectures as being a less positive example of teaching situations. Instead, they conveyed difficulties with adapting the teaching to the students’ needs and challenges in meeting individual students in a large group. Through discussions, preferably in small groups and seminars, the educators wanted the students to not only memorise facts, but also to reach a deeper understanding and be able to put theoretical knowledge into practice. The educators’ experiences of reflective discussions in smaller groups as positive learning examples in palliative care is confirmed by Hökkä et al., who state that teaching involving discussions has a positive impact on undergraduate nursing students’ attitudes toward death and caring for dying persons [18]. The importance of being able to formulate questions and discuss, in order to achieve long-term learning, is described by Land et al. The authors suggest that, when challenging existing knowledge through discussion, the chance of achieving new insights and knowledge increases [25]. In the present study, the educators described both having discussions where they as experts shared personal experiences from their work in palliative care as well as discussions between student peers at a similar level of knowledge.

A prerequisite for successful reflective discussions was, according to the educators in the study, that the students prepared themselves and were willing to share their experiences with their peers. Although most students were described as being motivated to participate in discussions about palliative care, some chose not to attend seminars that were not an examination and thus not mandatory. These students were described as missing the opportunity to participate in in-depth learning. Another prerequisite for successful reflections was a pragmatic and brave educator who felt secure enough to let the students’ discussions form the basis for learning. The educators described this pragmatic teaching approach as being challenging but rewarding. Problems occurred when the group dynamics affected the learning process negatively. They described these situations as being difficult to manage, and, when failing to steer the discussion in the right direction, they might even question their competence as teachers. Literature relating to the effect of group dynamics in nursing education is sparse, but a scoping review stated that there are five elements of group dynamics that influence small group learning in undergraduate students: engagement, openness, support, quality of communication, and style of dominant behaviour [26]. The authors suggest that educators, at the start of group activities, should inform the students about the elements that benefit group dynamics and, during learning activities, encourage behaviours that are conducive to learning. Students need to be aware of how they, through their behaviour and interaction, influence both their own learning and the learning of their peers [26].

Hökkä et al. show that there is a wide range of different education interventions that have positive effects on undergraduate nursing students’ knowledge of palliative care [18]. Among these, there are similarities, with examples found in the present study, such as simulation, visiting a crematorium, and group discussions. However, providing palliative care education in smaller groups, allowing time for reflection, and encountering distressing emotions requires resources, which might be limited, as palliative care is not always prioritised in curricula for Bachelor of Science nursing programmes [12]. Considering that palliative care is an approach that is relevant in almost all care contexts, for patients at all ages, and that the need for palliative care is increasing [2, 27], the subject should be prioritised in undergraduate nursing programmes, and all students should have the opportunity to prepare themselves for meeting and caring for dying persons and their relatives.

A strength of this study is that almost all eligible universities that provided nursing programmes participated, and educators willingly shared their experiences. There might be difficulties in interviewing by telephone as facial expressions or other reactions of the interviewee may be lost. On the other hand, follow-up questions for clarity are possible to ask and telephone interviews provide the opportunity to include participants from a distance [28], which in the present study enabled undergraduate nursing educators from each university across the country to participate.

The researchers in the present study have experience in palliative care, both as clinical nurses and educators. This can be considered a strength as it increases the possibilities of formulating relevant research questions. To manage the researchers’ preconceptions and avoid steering the research in any desired direction the interview guide was preformulated by the research group and contained open-ended questions. During the analysis results were continually discussed within the research group to do justice of the data.

As all interviewees described teaching situations, both successful and less successful, with a focus on emotional and existential aspects of palliative care, it may be wise to consider whether the data collection technique steered the answers in that direction. However, asking for one positive and one less positive teaching experience should act as a prompt to gather a wide range of experiences, not limit it down to one subject. The prominence of existential and emotional aspects of care in the educators’ narratives can be explained by the importance that educators attach to the subject and the effort that they make to incorporate it into their teaching practices.

It must be acknowledged that this study does not provide evidence about the effects of different types of pedagogical approaches, but the experiences of individuals. Future studies are needed to study the development of long-term knowledge through educational interventions.

## Conclusion

To prepare undergraduate nursing students for working in palliative care, education programmes should start by addressing the needs and resources of the students. Educators need to balance between letting the students seek out their own knowledge and providing them with information and guidance based on their expert knowledge. For students to reach a deeper understanding about palliative care as a subject, and of their own emotional reactions when encountering dying and grieving persons, educators should provide them with time for discussions and reflection, preferably in small groups. Performing this kind of dynamic teaching means being a facilitator rather than a classic lecturer. This requires educators to put their trust in both the students’ capabilities to take responsibility for their own learning and in their own abilities to cope with any situation that might occur. Having existing experience of working as nurses in palliative care seems to equip the educators with the necessary courage to follow and guide the students in a co-created learning process.

## Data Availability

The datasets used and/or analysed during the current study are available from the corresponding author on reasonable request.
